# Mitoxantrone hydrochloride injection guided sentinel lymph node biopsy in cN0 BRAF V600E-mutated papillary thyroid carcinoma: a feasibility study

**DOI:** 10.3389/fendo.2025.1732034

**Published:** 2025-12-11

**Authors:** Zengwei Li, Chunyang Li, Jingxian Li, Tingfei Song, Xiaojuan Men, Yonghui Wang

**Affiliations:** 1 School of Clinical Medicine, Shandong Second Medical University, Weifang, Shandong, China; 2 Department of Thyroid and Breast Surgery, Anqiu People’s Hospital, Weifang, Shandong, China; 3 Department of Thyroid Surgery, Weifang People’s Hospital, Weifang, Shandong, China

**Keywords:** mitoxantrone hydrochloride injection, sentinel lymph node biopsy, papillary thyroid carcinoma, BRAF V600E mutation, prophylactic central neck dissection

## Abstract

**Background:**

The BRAF V600E mutation in papillary thyroid carcinoma (PTC) is linked to aggressive behavior and frequent occult central lymph node metastases (CLNM), posing a surgical dilemma in clinically node-negative (cN0) patients. Sentinel lymph node biopsy (SLNB) could enable selective intervention, but its accuracy depends on tracer performance. We investigated the utility of SLNB using mitoxantrone hydrochloride injection (MHI) as a novel lymphatic tracer to determine its feasibility and diagnostic performance for occult metastases in patients with cN0, BRAF V600E–mutated, unilateral PTC.

**Methods:**

48 patients with cN0, BRAF V600E-mutated unilateral PTC underwent intraoperative SLNB using MHI, followed by ipsilateral lobectomy with isthmusectomy and prophylactic central neck dissection (PCND). The diagnostic accuracy of SLNB was evaluated by comparing its results with the final histological results from PCND serving as the gold standard. Short-term postoperative complications were recorded to assess the safety of the procedure.

**Results:**

Sentinel lymph nodes (SLNs) were identified in 46 out of 48 patients (95.8%), with 62 SLNs retrieved, most commonly in the peritracheal region (93.5%). Intraoperative frozen section analysis identified 21 patients with positive SLNs and 25 with negative SLNs. Final pathology confirmed CLNM in 23 patients (47.9%). The sensitivity, specificity, negative predictive value (NPV), and positive predictive value (PPV) of SLNB for detecting CLNM were 91.3%, 100%, 92.6%, and 100%, respectively. Postoperatively, two patients (4.2%) experienced transient vocal cord palsy, with complete resolution within one week. No cases of parathyroid injury or removal were observed, and there were no instances of transient hypoparathyroidism or permanent complications.

**Conclusion:**

In patients with cN0, BRAF V600E–mutated PTC, SLNB using mitoxantrone hydrochloride injection is technically feasible and demonstrates high sensitivity and specificity. This approach may help identify individuals with occult central compartment metastases, thereby potentially avoiding unnecessary PCND in those with negative SLNs.

## Introduction

Thyroid carcinoma represents the most common malignancy of the endocrine system, with its global incidence continuing to rise in recent years. Thyroid cancers are classified into several types, including differentiated thyroid carcinoma (such as papillary and follicular carcinoma), medullary thyroid carcinoma, and anaplastic carcinoma. Among these, papillary thyroid carcinoma (PTC) is the most prevalent. The prognosis for most PTC patients is favorable, with a 10-year survival rate exceeding 90% (1). However, a subset of cases exhibits more aggressive biological behavior and a higher risk of lymph node metastasis.

Molecular biological studies have demonstrated that the development of most PTCs (90%) is associated with activation of the mitogen-activated protein kinase (MAPK) pathway, primarily driven by mutually exclusive mutations in oncogenes such as BRAF or RAS (2, 3). Among these, the BRAF V600E mutation, the most frequent genetic alteration in PTC, has been extensively corroborated to correlate with enhanced tumor aggressiveness and increased risk of lymph node metastasis (4, 5). Central lymph node metastasis (CLNM) is the most common form of occult metastasis in these patients, with a particularly high incidence observed in those harboring the BRAF V600E mutation (6). Currently, controversy persists regarding whether prophylactic central neck dissection (PCND) should be performed for clinically node-negative (cN0) BRAF V600E mutation-positive PTC patients (7). Notably, the American Thyroid Association (ATA) guidelines do not explicitly recommend expanding surgical scope based solely on the presence of mutation (1).

Therefore, this study focuses on patients with cN0, BRAF V600E-mutated, unilateral PTC. It aims to systematically evaluate the feasibility of SLNB using mitoxantrone hydrochloride injection as a novel lymphatic tracer and to thoroughly investigate its diagnostic value for detecting occult central compartment metastases, along with the safety profile regarding postoperative complications. As all patients subsequently underwent PCND, the postoperative complications recorded reflect the combined safety profile of SLNB followed by PCND, allowing for observation of the technique’s integration into surgical practice.

## Materials and methods

### Patients

**Figure 1:**
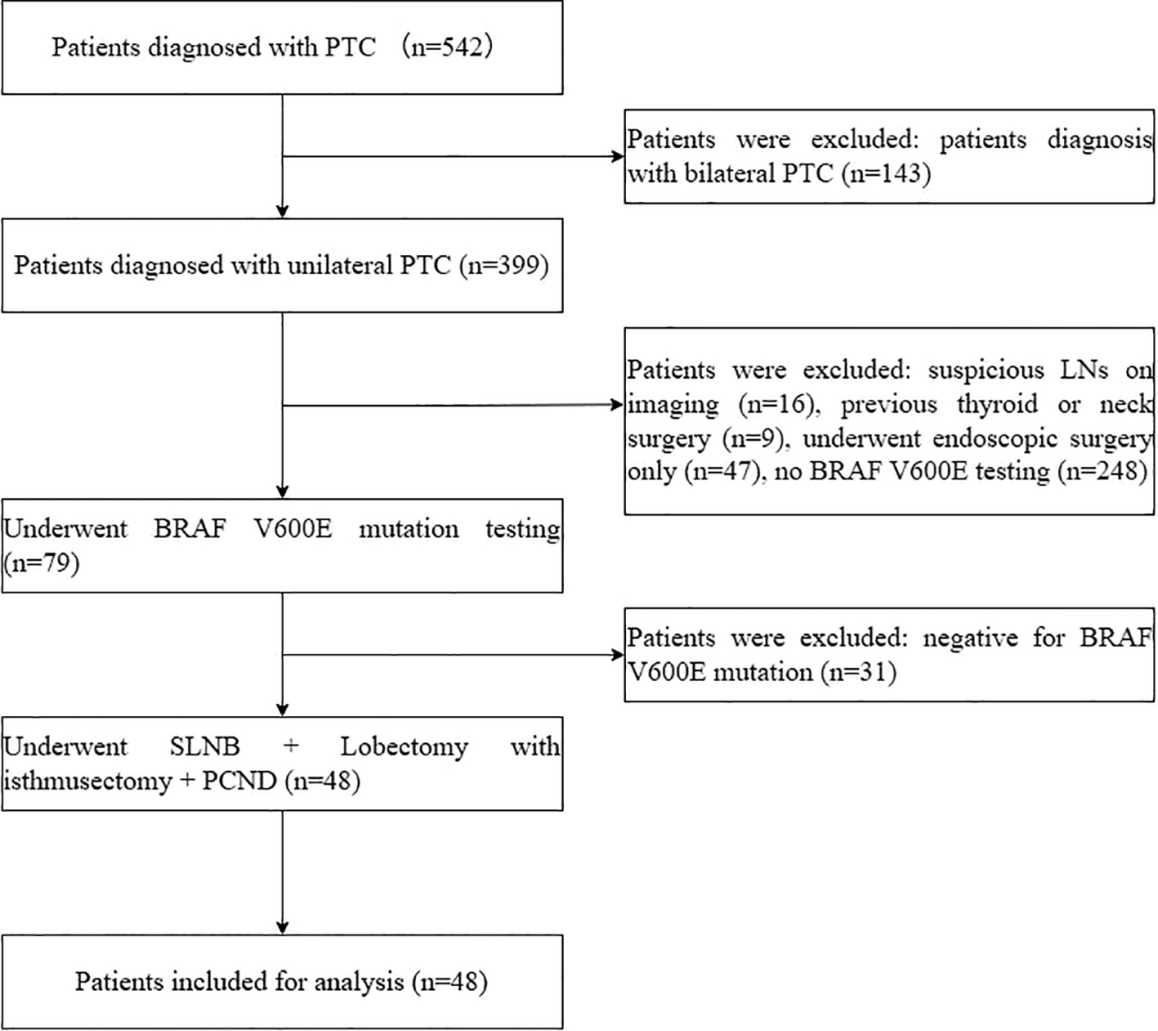
A CONSORT diagram showing the patient inclusion and exclusion criteria.

This retrospective study enrolled a cohort of 48 patients with unilateral PTC diagnosed by ultrasonography or fine-needle aspiration (FNA) between October 2024 and May 2025. BRAF V600E mutation positivity was confirmed preoperatively in fine-needle aspiration specimens using quantitative real-time PCR. Patients with BRAF V600E mutation were selectively included to focus on a population at higher risk of occult central lymph node metastasis, thereby allowing a more precise evaluation of the clinical utility of SLNB in this subgroup. Exclusion criteria consisted of previous thyroid or neck surgery, palpable or imaging-detected lymph node involvement (by ultrasound or CT), or other types of thyroid malignancy. A final cohort of 48 patients who met the inclusion criteria was enrolled; the patient selection process is summarized in **Figure 1**. All patients underwent conventional surgery plus PCND following SLNB. The surgical approach was not randomized and was determined based on surgical team consensus and patient preference regarding the extent of resection. Written informed consent was obtained from all patients and their families after detailed explanation of the SLNB procedure and thorough discussion of surgical risks and benefits.

### Surgical procedure

A layered incision and dissection were performed to routinely expose the ventral capsule of the thyroid gland. The dissection typically did not extend beyond the middle and lateral thirds of the gland to avoid disrupting the perithyroid lymphatic network through excessive mobilization. After exposing the thyroid, 0.5 mL of mitoxantrone hydrochloride injection (MHI) at a concentration of 0.5 mg/mL was administered using a 1 mL syringe. Depending on the gland size, the tracer was injected superficially into the thyroid parenchyma at three points (superior, middle, and inferior) around the perimeter of the tumor, at a depth of approximately 5 mm, taking care to avoid intravascular injection. Immediately after injection, gentle pressure was applied to the injection sites with sterile gauze for 1 minute to minimize tracer leakage. Any leakage was promptly and thoroughly wiped with gauze to minimize contamination of the surgical field and avoid interference with subsequent identification. After a waiting period of 5 minutes, allowing the lymphatic vessels and nodes to exhibit clear and stable blue staining, all blue-stained SLNs were meticulously dissected. These SLNs were separately sent for intraoperative frozen section pathological examination. An SLN was defined as the first lymph node to show blue staining or a node directly drained by a blue-stained lymphatic vessel. In cases of atypical staining patterns or multiple lymphatic drainage pathways, the lymph node located closer to the gland, staining earlier and more intensely, was identified as the SLN. Subsequently, conventional lobectomy and isthmusectomy were performed. This was followed by comprehensive dissection of the remaining central compartment lymph nodes. All dissected lymph nodes from the PCND were submitted en bloc for routine pathological examination. The pathological results from the PCND served as the gold standard for diagnosing CLNM.

All resected SLNs and PCND specimens were fixed in 10% formalin, embedded in paraffin, and serially sectioned at a thickness of 2-3 μm. Sections were stained with hematoxylin and eosin (H&E). All pathological slides were independently reviewed by two senior pathologists who were blinded to the SLNB results. In cases of diagnostic disagreement, a third senior pathologist was consulted to achieve a final consensus.

**Figure 2:**
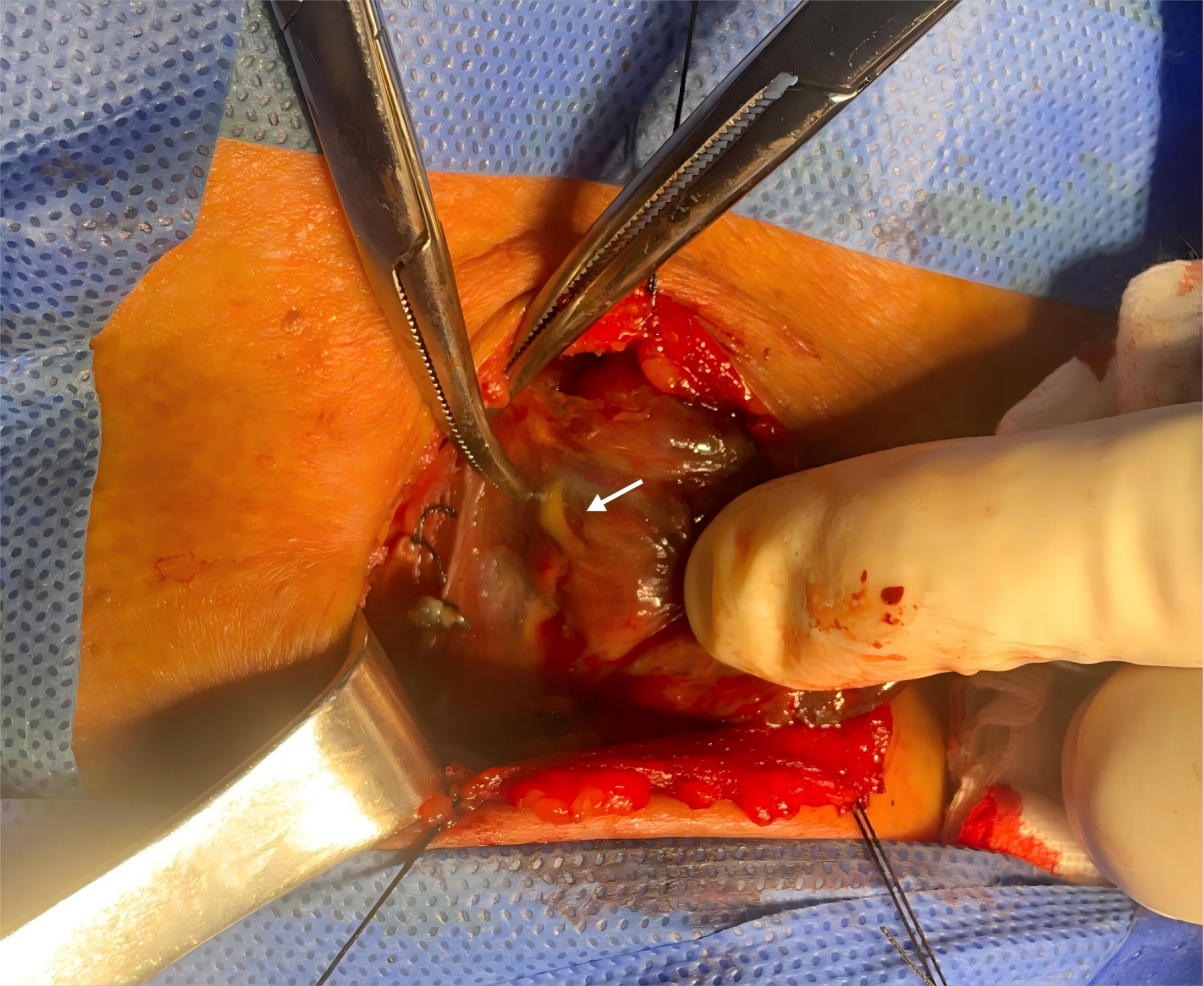
Negative staining of the parathyroid gland. Arrow indicates the non-blue-stained parathyroid gland.

Concurrently, the “negative staining” technique, which identifies tissue areas not stained by MHI, was utilized to aid in the identification and meticulous preservation of the parathyroid glands and recurrent laryngeal nerves. A representative image of negative staining for a parathyroid gland is shown in **Figure 2**. To minimize procedural variability, all steps (including MHI injection, SLN mapping, recurrent laryngeal nerve exposure, and regional lymph node dissection) were performed by a single senior surgeon with over 15 years of specialized experience in thyroid surgery.

### Statistical analysis

Data analysis was performed using GraphPad Prism version 10. Categorical variables were compared using the Chi-square test or Fisher’s exact test, as appropriate. Continuous variables were assessed for normality with the Shapiro–Wilk test and subsequently compared using the independent samples t-test or Welch’s t-test. The detection rate, sensitivity, specificity, positive predictive value (PPV), and negative predictive value (NPV) of SLNB were calculated along with their 95% confidence intervals, estimated by the Wilson/Brown method. Patients were stratified into CLNM-positive and CLNM-negative groups based on final pathological results for subgroup analysis, in which baseline clinicopathological characteristics were compared. All tests were two-sided, and a P-value of less than 0.05 was considered statistically significant. Data on postoperative complications were obtained from follow-up records.

## Results

We enrolled 48 consecutive patients with BRAF V600E–mutated, cN0 unilateral PTC. None of the patients exhibited clinical or radiological features suggestive of lymph node metastasis. The mean age was 45.6 ± 11.8 years (range, 19–69). The mean tumor size was 0.75 ± 0.43 cm, with 40 cases (83.3%) classified as microcarcinomas (≤1.0 cm). A solitary tumor focus was present in 42 patients (87.5%), while unilateral multifocality was observed in 6 patients (12.5%). Extrathyroidal extension was identified in 26 patients (54.2%). Concurrent Hashimoto’s thyroiditis was documented in 7 patients (14.6%). Tumor location was distributed as follows: upper pole in 13 patients (27.1%), middle pole in 24 patients (50.0%), and lower pole in 11 patients (23.0%). No thyroid fibrosis was detected on preoperative imaging or during intraoperative evaluation. The baseline demographic and clinicopathological characteristics of the patients are summarized in **Table 1**.

**Table 1:** Patient demographics and tumor characteristics (n=48).

Characteristic	Value
Sex	
Male	8
Female	40
Age (years)	
Mean ± SD (range)	45.6 ± 11.8 (19-69)
Group (aged<55y vs aged≧55y)	33/15
Primary tumor	
Size, cm, mean ± SD (range)	0.75 ± 00.43 (0.1-2.0)
Groups: ≤1cm vs>1cm	40/8
Location	
Upper portion	13
Middle portion	24
Lower portion	11
Concomitant Hashimoto’s thyroiditis	7
Unifocal	42
Multifocal unilateral	6
Extracapsular invasion	26
TNM classification	
I	36
II	9
III	3

**Figure 3:**
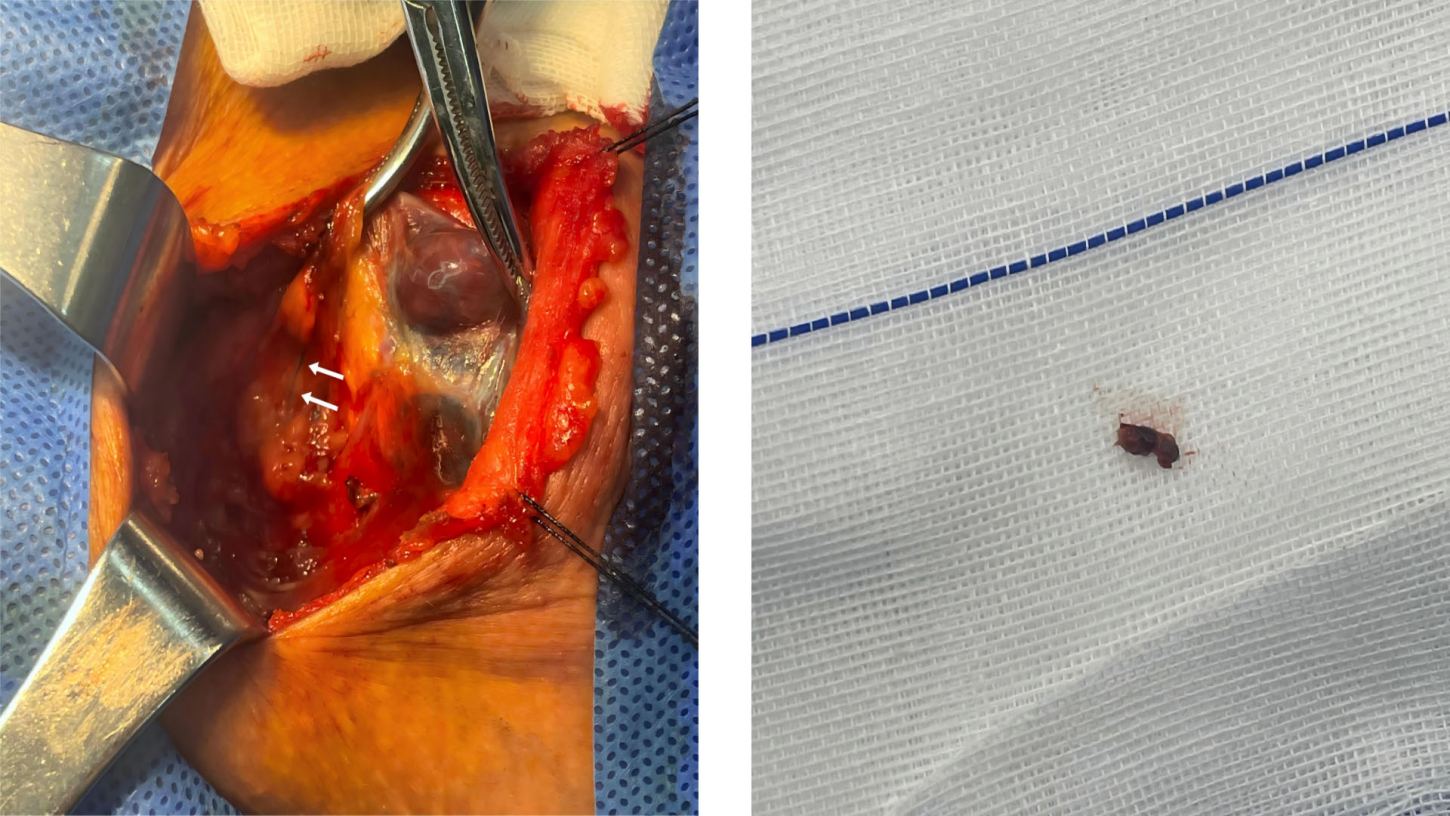
Peritracheal blue-stained lymphatic vessel and lymph nodes. Arrow indicates the blue-stained lymphatic vessel in the central compartment.

**Figure 4:**
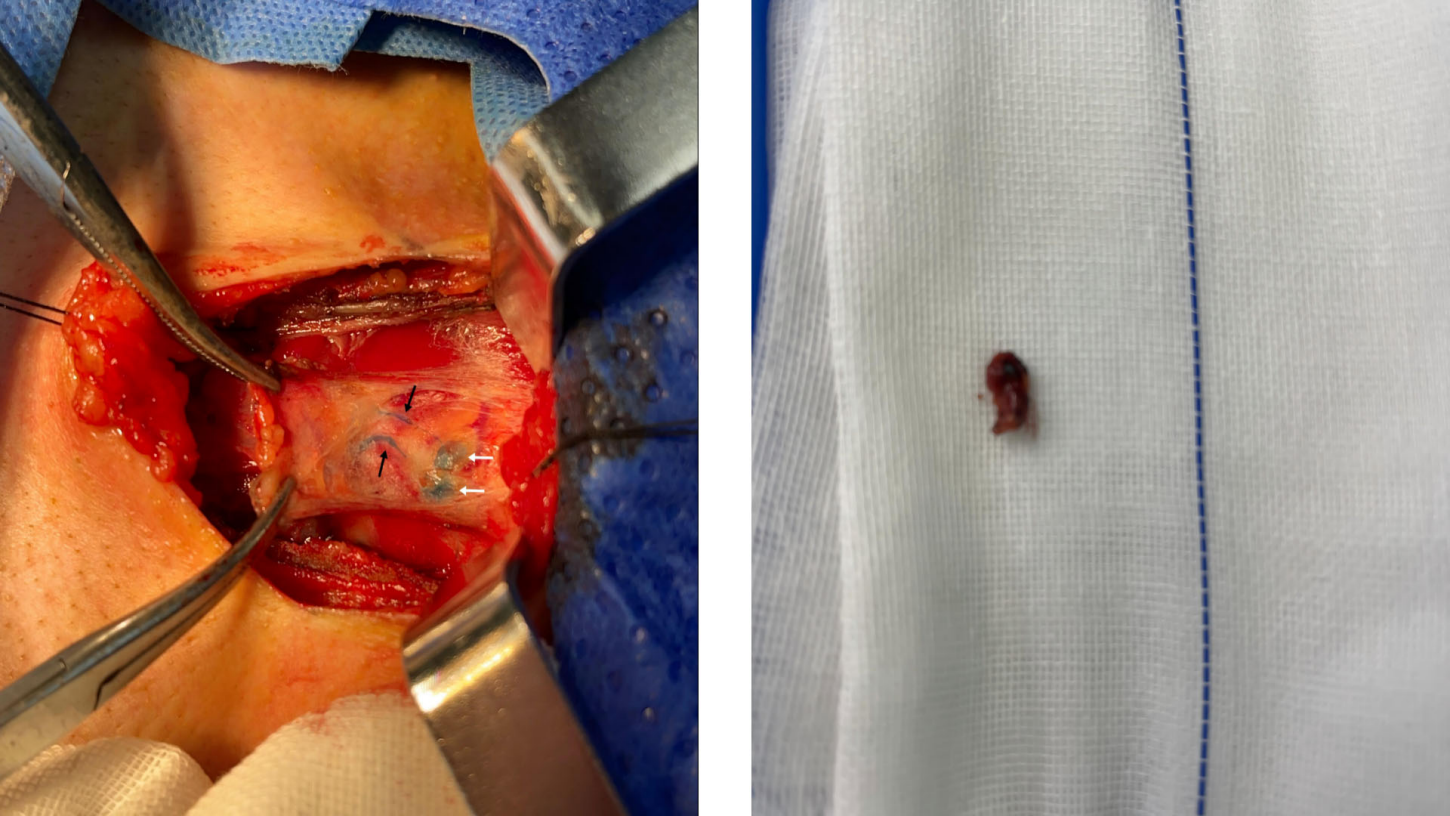
Prelaryngeal blue-stained lymph vessels and lymph nodes. The black and white arrows indicate the blue-stained prelaryngeal lymphatic vessels and lymph nodes, respectively.

All patients underwent ipsilateral lobectomy with isthmusectomy, concurrently with both PCND and SLNB. SLNs were successfully identified via blue-stained lymphatic channels in 46 patients (95.8%), yielding a total of 62 blue-stained SLNs, with a mean of 1.3 ± 0.6 SLNs per patient. In 2 patients (4.2%), no SLN could be visualized. The SLNs were predominantly located in the peritracheal area (58 nodes, 93.5%), with the remainder found in the prelaryngeal area (4 nodes, 6.5%). Representative images of the blue-stained lymphatic vessels and nodes in the peritracheal and prelaryngeal areas are shown in **Figures 3**, **4**, respectively.

Among the 46 patients with successful SLN mapping, intraoperative frozen section analysis revealed metastatic involvement in 21 SLNs and was negative in 25. Final histopathological examination confirmed the presence of central lymph node metastases in all 21 patients with positive SLNs; among these, 17 patients had additional metastatic nodes found within the central compartment upon completion PCND. Conversely, no metastases were detected in the 25 patients with negative SLNs. However, final pathology confirmed central compartment metastases in 2 patients whose SLNs were intraoperatively reported as negative (**Table 2**). Based on the final histopathological results, the sensitivity, specificity, false-negative rate, false-positive rate, NPV, and PPV of SLNB for detecting central lymph node metastasis were 91.3%, 100%, 8.7%, 0%, 92.6%, and 100%, respectively. The corresponding 95% confidence intervals are presented in **Table 3**. The difference was statistically significant (P < 0.0001). Among the two false-negative cases, Case 1 was a 48-year-old female with a tumor located in the upper pole of the left lobe (size 0.9 cm) and concomitant Hashimoto’s thyroiditis. Case 2 was a 37-year-old male with a tumor in the upper pole of the right lobe (size 0.8 cm) and the presence of extrathyroidal extension. Final pathology in both cases revealed one lymph node with micrometastasis. It is plausible that the false-negative findings resulted from micrometastatic deposits that were too small to be reliably detected by intraoperative frozen section. Moreover, a high level of concordance was observed between the final paraffin section diagnoses and the intraoperative frozen section findings for all SLNs. Independent assessments by two pathologists reached consensus in all specimens.

**Table 2:** Feasibility of sentinel lymph node biopsy in the central compartment.

Diagnostic performance	Pathology		
	Positive	Negative	Total
SLN	21 (TP)	0 (FP)	21
Non-SLN	2 (FN)	25 (TN)	27
Total	23	25	48

TP, true positive; FP, false positive; FN, false negative; TN, true negative.

**Table 3:** Results of SLNB using mitoxantrone hydrochloride injection in the central compartment.

Characteristic	No. cases	Percentage of total	95%CI
Detection rate	46/48	96%	
Sensitivity (TP)	21/23	91.3%	73.2-98.5
Specificity (TN)	25/25	100%	86.7-100
PPV	21/21	100%	84.5-100
NPV	25/27	92.6%	76.6-98.7

SLNB, sentinel lymph node biopsy; CI, confidence interval; TP, true positive; TN, true negative; PPV, positive predictive value; NPV, negative predictive value.

To further evaluate the diagnostic performance of SLNB, clinicopathological characteristics were compared between patients with and without CLNM (**Table 4**). The CLNM-positive group had significantly larger tumor size (1.00 ± 0.48 cm vs. 0.54 ± 0.23 cm, P < 0.001) and a higher rate of extrathyroidal extension (71.4% vs. 44.0%, P = 0.06). No significant differences were observed between the two groups in terms of age, sex, tumor multifocality, or the incidence of Hashimoto’s thyroiditis (all P > 0.05).

**Table 4:** Comparison of clinicopathological characteristics between CLNM-positive and CLNM-negative patients.

Characteristic	CLNM-Positive (n=21)	CLNM-Negative (n=25)	P
Age (years), Mean ± SD	46.24 ± 11.97	45.72 ± 12.14	0.89
Sex, Female, n (%)	15 (71.4)	23 (92.0)	0.12
Tumor Size (cm), Mean ± SD	1.00 ± 0.48	0.54 ± 0.23	<0.001
Tumor Multifocality, n (%)	4 (19.0)	2 (8)	0.39
Extrathyroidal Extension, n (%)	15 (71.4)	11 (44)	0.06
Concomitant Hashimoto’s Thyroiditis, n (%)	4 (19.0)	3 (12)	0.69

A P value less than 0.05 was considered statistically significant.

CLNM, central lymph node metastasis; SD, standard deviation.

No instances of parathyroid gland injury or inadvertent resection due to blue staining were identified during surgery. Postoperatively, transient vocal cord palsy occurred in 2 patients (4.2%), with function recovering fully within one week. No cases of permanent recurrent laryngeal nerve palsy or transient hypoparathyroidism were recorded. Four patients were lost to follow-up within 4 months post-surgery. The remaining 44 patients were followed for a mean of 7 months (range: 5–12), during which no cases of locoregional recurrence or distant metastasis were observed. While this duration is sufficient to assess short-term complications and the technical feasibility of the procedure, it is insufficient to evaluate long-term oncological outcomes such as locoregional recurrence or disease-free survival.

## Discussion

The BRAF V600E mutation represents one of the most frequent genetic alterations in PTC. It drives tumorigenesis by constitutively activating BRAF kinase activity, thereby promoting cell proliferation, aberrant differentiation, and apoptosis evasion. This mutation potently enhances MAPK signaling pathway activity, which is associated with increased tumor aggressiveness, abnormal thyroid cell proliferation, and a strong correlation with higher rates of lymph node metastasis and elevated recurrence risk (4, 5, 8). Multiple studies, including reports by Tabriz et al., have confirmed the association of the BRAF V600E mutation with more aggressive clinicopathological features, such as extrathyroidal extension, multifocality, and a higher incidence of CLNM (9). Although its relationship with disease recurrence remains somewhat controversial (10), this mutation unequivocally signifies a greater potential for local invasion, particularly manifesting in a high rate of occult CLNM. While the BRAF V600E mutation has been historically associated with more aggressive tumor features, its independent clinical utility as a definitive prognostic factor is actively debated. Furthermore, some studies suggest that a higher mutant allele frequency of BRAF V600E may correlate with more aggressive tumor behavior (11). The combined assessment of BRAF V600E mutation status and allele frequency could inform surgical decision-making by identifying tumors with aggressive potential, thereby underscoring its value as a prognostic biomarker for determining the extent of resection and lymph node dissection.

Despite the established link between BRAF V600E mutation and increased risk of nodal metastasis, the routine performance of PCND in patients with cN0 PTC remains a subject of debate (12, 13). Proponents argue that PCND enables precise pathological staging and guides subsequent therapeutic decisions. Zocchi et al. indicates that PCND can detect occult CLNM in 30.6% of patients, leading to restaging and adjuvant therapy in 12.5% (14); this diagnostic and therapeutic rationale is further strengthened Barczyński et al., which demonstrated that RAI treatment individualized based on pCND findings significantly improved 10-year disease-specific survival (15). However, several studies have failed to demonstrate a significant improvement in long-term disease-free survival attributable to PCND, while concurrently highlighting its association with increased postoperative complication rates due to the more extensive dissection (7, 16). Studies by Dobrinja and Dismukes et al. emphasized that the PCND group experienced a significantly higher complication rate without a corresponding reduction in tumor recurrence, leading them to recommend avoiding PCND in cN0 PTC and reserving it for “high-risk” patients to mitigate locoregional recurrence (17, 18). While the 10-year randomized controlled trial by Piermarco Papini et al. rightly questions the routine use of PCND in cN0 PTC by showing no overall benefit, its conclusions are primarily applicable to the low-risk population of small tumors (13). Thus, the critical question becomes how to definitively identify those patients with occult disease preoperatively. Our study addresses this by evaluating an SLNB strategy designed to personalize the decision for dissection, specifically targeting those patients at higher risk who stand to gain the most.

We performed PCND to validate the accuracy of the tracer. Using MHI as a lymphatic tracer, a SLN identification rate of 95.8% was achieved, demonstrating the technical feasibility of the procedure. Regarding diagnostic performance, SLNB showed a sensitivity of 91.3% and a NPV of 92.6% for detecting CLNM, indicating its high reliability in ruling out central compartment metastases and its potential to avoid unnecessary PCND in patients with SLN-negative findings. Although the specificity and PPV were both 100% in this study, suggesting no false-positive cases, it is noteworthy that two false-negative cases occurred, resulting in a false-negative rate of 8.7%. This indicates that the technique still carries a certain risk of missed diagnoses.

The accuracy of SLNB depends on consistent lymphatic drainage, which can be altered or disrupted by various factors. This study documented and analyzed clinical variables potentially affecting lymphatic drainage. First, Hashimoto’s thyroiditis, as a chronic inflammatory disease, may cause structural changes or obstruction of lymphatic vessels, potentially leading to incomplete tracer uptake or drainage to non-typical lymph nodes. In this study, one of the false-negative cases was complicated by Hashimoto’s thyroiditis, which may have contributed to the failure of the SLN to capture the micro-metastasis. Second, specific tumor locations, such as the upper pole, may involve more complex lymphatic drainage pathways, occasionally including the possibility of “skip metastases” directly draining to the lateral neck compartment. This increases the risk that SLNB may not fully represent the central compartment metastatic status. Furthermore, extensive extrathyroidal extension may disrupt the normal lymphatic network, potentially causing disorganized tracer drainage.

In addition to the above lymphatic drainage factors, subgroup analysis revealed that CLNM was significantly associated with larger tumor size and a higher rate of extrathyroidal extension, consistent with previous reports on the aggressive features of BRAF V600E-mutated PTC (9). This finding indicates that SLNB maintained a high sensitivity of 91.3% even within this subgroup of patients with clear high-risk characteristics. In other words, the technique rarely missed metastases even in the subgroup more prone to metastasis. More importantly, this analysis helps to reasonably explain the two false-negative cases. As described in the Results, both cases involved micro-metastases, which were likely below the detection threshold of intraoperative frozen section analysis. Despite these instances, SLNB achieved an overall NPV of 92.6% across the entire cohort. This implies that for patients with a negative SLNB result, omitting PCND in the short term is a reliable approach.

The lymphatic tracer used in this study, MHI, leverages its self-assembling nanocrystal structure, endowing it with a strong affinity for the lymphatic system and excellent intraoperative visibility (19). Compared to other tracers—such as methylene blue, which suffers from rapid diffusion and persistent tissue staining; radiocolloids, which require specialized equipment and involve radiation exposure; as well as carbon nanoparticles, which may cause lymphatic blockage or contamination of the surgical field—MHI is engineered with a particle size that prevents intravascular penetration while ensuring stable retention within lymph nodes, resulting in sustained and clear blue staining. A multicenter, self-controlled clinical trial in early breast cancer patients reported a relative detection rate of 97.31% for MHI, demonstrating non-inferiority compared to radioactive technetium-99m, with a high concordance rate of 79.69% between the two methods. This evidence strongly supports the superior detection capability of MHI (20). In the present study, MHI achieved an SLN detection rate of 95.8%, demonstrating reliable targeting performance. This result is consistent with preliminary reports in fields such as breast cancer, and supports MHI as a preliminary and valuable diagnostic tool for sentinel lymph node identification in thyroid carcinoma.

Beyond its tracing performance, the combination of MHI with the “negative staining” technique enhanced the intraoperative identification of parathyroid glands, thereby reducing the risk of their injury or accidental resection. Preserving parathyroid function remains a critical challenge in central neck surgery. CND has been established as a significant and independent risk factor for incidental parathyroidectomy (IP). Evidence confirms that patients undergoing CND face a substantially elevated risk of IP, with multivariate analyses reporting odds ratios of 2.68 for prophylactic and 4.44 for therapeutic CND (21). Importantly, IP itself is significantly associated with an increased risk of both transient and permanent hypoparathyroidism postoperatively (22). In contrast, by precisely mapping SLNs and utilizing the “negative staining “ principle to facilitate the reverse identification of parathyroid glands, our study observed no instances of accidental parathyroid resection or injury attributable to the blue stain, and no cases of transient hypoparathyroidism occurred postoperatively. This highlights the feasibility and advantage of MHI in effectively balancing precise lymph node tracing with the crucial goal of parathyroid preservation.

This study has several limitations. First, its single-center, retrospective design and limited sample size may introduce selection bias. Second, although all surgical procedures and SLNB identifications were performed by a single surgeon to ensure technical consistency, the results lack an evaluation of inter-operator reproducibility. Third, the absence of a control group using other tracers, such as methylene blue or carbon nanoparticles, means that the relative advantages of MHI cannot be definitively established. Finally, the mean follow-up period of only seven months is insufficient to assess long-term local tumor control rates or late complications.

In conclusion, the use of MHI for SLNB in patients with cN0 BRAF V600E-mutated unilateral PTC is both feasible and effective, demonstrating high sensitivity and specificity. This tracer facilitates the identification of patients with occult central compartment metastases, thereby providing a rationale for selective central neck dissection. By avoiding unnecessary PCND and aiding parathyroid preservation in SLN-negative patients, this approach could reduce surgery-related morbidity. Future studies should involve larger, multicenter prospective cohorts, include direct comparisons with other tracers, and incorporate cost-effectiveness analyses to further validate the clinical value and long-term outcomes of this technique.

## Data Availability

The raw data supporting the conclusions of this article will be made available by the authors, without undue reservation.
